# The impact of COVID-19 on the quality of life of older adults: a cross-sectional study in Athens, Greece

**DOI:** 10.1192/j.eurpsy.2022.1225

**Published:** 2022-09-01

**Authors:** K. Argyropoulos, Z. Tagkalaki, A. Argyropoulou, D. Avramidis, E. Jelastopulu

**Affiliations:** 1 School of Medicine, University of Patras, Public Health, Rio, Greece; 2 Private practice, Internal Medicine, Athens, Greece

**Keywords:** GDS-15, Elderly, WHO-5, Covid-19

## Abstract

**Introduction:**

The COVID-19 has affected both physical and mental health of the elderly.

**Objectives:**

The purpose of the present study was to estimate the impact of the second lockdown in Greece, on both quality of life and mental health in older people.

**Methods:**

A cross sectional study was conducted among older adults who visited a primary care physician, from 1^st^ of March to April 30^th^. An anonymous questionnaire was administered to collect basic sociodemographic data and implementation of hygiene precaution measures. The 5-item World Health Organization Well-Being Index (WHO-5) to measure well-being, the Generalized Anxiety Disorder Assessment (GAD-7) instrument was used to assess the anxiety levels and Geriatric Depression Scale (GDS-15) depressive symptoms of the responders, respectively. Statistical analysis was performed with SPSS v.24.0

**Results:**

222 elderly took part in the study. 62.6% were female. According to the WHO-5, 37.4% present poor quality of life. GDS-15 reveals that 70.7% of the participants screened positive for moderate depression and 1.8% with severe symptoms. GAD-7 results estimated 32.9% of the participants to suffer from serious anxiety disorder and 37.4% from moderate. GAD-7 and GDS-15 were strongly associated (p <0.05) with female gender, low educational level and with comorbidities (coronary disease, diabetes mellitus and skeletomuscular diseases). Health precaution measures were negative correlated with mental health of the elderly. However, in participants with frequent contact with family and friends, lower anxiety levels were detected.

**Conclusions:**

Our results highlight that older adult has experienced negative impact on both quality of life and mental health during 2^nd^ Covid-19 lockdown in Greece.

**Disclosure:**

No significant relationships.

